# Application and Technical Principles of Catheter High-Frequency Jet Ventilation

**DOI:** 10.3390/arm91040022

**Published:** 2023-06-27

**Authors:** Peter Musil, Stefan Harsanyi, Pavol Torok, Monika Paulikova, Didier Moens, Ladislav Kalas, Peter Kalas

**Affiliations:** 1 Faculty of Medicine, Comenius University in Bratislava, 811 08 Bratislava, Slovakia; stefan.harsanyi@fmed.uniba.sk; 2 Department of Anesthesiology and Intensive Care Medicine, VÚSCH a.s. Košice, 040 11 Košice, Slovakia; torokpavol@gmail.com (P.T.);; 3 Kalas Medical, Slovenských Partizánov 1130, 017 01 Považská Bystrica, Slovakiakalas@kalas.sk (L.K.);

**Keywords:** catheter high-frequency jet ventilation, C-HFJV, artificial lung ventilation, ALV, physical principles

## Abstract

**Highlights:**

Catheter methods of high-frequency jet ventilation (HFJV) have proven to be very effective artificial lung ventilation (ALV) methods in technical experimental conditions as well as in clinical applications.

**What are the main findings?**

- Explanation of potentially optimal techniques for catheter high-frequency jet ventilation (C-HFJV) applications.
- Assessment and clarification of application methodologies of C-HFJV ventilation, its biophysical principles of function, and solutions to prevent volutrauma and barotrauma.

**What is the implication of the main finding?**

- The positive effects of C-HFJV, even with complications, such as bacterial or viral pneumonia, including COVID-19.
- The method of double-lumen catheter ventilation significantly contributes to clinical practice.

**Abstract:**

The aim of this publication is to analyze the topic of high-frequency jet ventilation (HFJV), namely catheter HFJV (C-HFJV), from a mathematical–physical as well as a clinical point of view. There are known issues with applying anesthesia and artificial lung ventilation (ALV) during surgical procedures in the upper airways, e.g., during bronchoscopy or tracheostomy. The principles, advantages, and disadvantages of HFJV are discussed in context with basic physical principles to clarify the proper use of this method. The basic technical principles of catheter construction, as well as its functional properties from a biophysical point of view, are introduced. Also, the placement of the catheter in the airways, the set-up of the HFJV ventilator, and the indications as well as the risks and contraindications of the use of C-HFJV are analyzed. This leads to the explanation of potentially optimal techniques for C-HFJV applications. In this article, we present the positive effects of C-HFJV even with complications such as bacterial or viral pneumonia, including COVID-19. In conclusion, we offer recommendations for clinical practice obtained from a literature review and from our rich clinical experience.

## 1. Introduction

Interventions in otorhinolaryngology (ORL), tracheal surgery, intensive care medicine, and thoracic surgery represent a specific situation for surgeons and anesthesiologists. Unlike in other surgical fields, the respiratory tract is a place of interest for both specialists. The requirements of the performing physician for optimal access to the operative field often conflict with the anesthesiologist’s requirements to ensure patency of the airways and ventilation. Similarly, with tracheostomy and rigid or flexible bronchoscopy, the specialist’s requirements are fairly specific [1].

The choice of anesthesiologic approach and artificial lung ventilation (ALV) type in patients with airway or lung pathology, be it pneumonia or COVID-19-associated ARDS (CARDS), largely depends on the pathological process in the respiratory tract, as well as on the traumatic changes in the airways, associated lung diseases (including COVID-19), and especially in oncologic patients when the airways are obstructed by a growing tumor. In addition, patients with bronchopneumonia have a risk of worsening the gas exchange in the lungs. In order to find an optimal solution, many different techniques were unsuccessfully used in the past: spontaneous ventilation and topical anesthesia, apneic oxygenation, intermittent apnea, and intubation with a thin endotracheal cannula. In the 1970s, high-frequency jet ventilation (HFJV) was developed [2] to reduce the risk of the aspiration of debris and blood and minimize vocal cord movements. Currently, the method of catheter high-frequency jet ventilation (C-HFJV) is often used.

Most of the studies so far have been focused on evaluating the effectiveness and safety of tracheotomies, whether according to Fantoni or puncture dilatation tracheotomy, bronchoscopy, or in patients with pneumonia. Studies have also focused on evaluating patients with tumorous changes in the upper airways or with traumatic changes in the trachea and bronchi. In this article, we present the positive effects of C-HFJV even in cases complicated by bacterial or viral pneumonia, including COVID-19. Our aim was to assess and clarify the application methodologies of C-HFJV, its biophysical principles of function, as well as solutions to prevent barotrauma and volutrauma. We offer recommendations for clinical practice obtained from a literature review and our rich clinical experience.

## 2. High-Frequency Jet Ventilation

The HFJV method is based on the application of small volumes of gas under high pressure through a specially adapted cannula (catheter), without sealing the airways with a balloon, thus leaving the airways with free communication with the surrounding environment or using a special multi-nozzle jet injector (MNJI) [3]. In order to achieve adequate minute ventilation, it is necessary to ensure a high respiratory frequency with small applied volumes of gas [3,4]. The advantages and disadvantages of HFJV are presented in Table 1.

Types of HFV Techniques:

1. HFPPV (high-frequency positive pressure ventilation)–high-frequency ventilation with positive pressure using a frequency of up to 200 cycles/min;
2. HFJV (high-frequency jet ventilation)–high-frequency ventilation using a nozzle to generate a jet stream with a frequency of up to 600 cycles/min;
3. HFFI (high-frequency flow interruption)–also known as HFPV (high-frequency percussive ventilation) is characterized by short bursts of gas delivered directly into the ventilator circuit without the injector cannula used in HFJV [5];
4. HFO (high-frequency oscillation)–high-frequency ventilation that uses very small tidal volumes often lower than the dead space, with a frequency normally above 400 cycles/min, often reaching as much as 600–900 cycles/min [6].

From the technical implementation point of view, the ventilators are divided into:

(a) classic ventilator with a higher breathing frequency (HFPPV);
(b) ventilator with nozzle-type injector and receiving channel (HFJV);
(c) special HF ventilators using piston, bubble, and membrane, to generate oscillation (HFO).

Methods of application–interface:

From the point of view of the ventilation circuit closure to the atmosphere, HFV can be divided into closed systems (i.e., hermetically sealed circuit with cuffed endotracheal tube) and open systems (non-hermetic circuit with an unsealed endotracheal tube or with a bi-nasal cannula).

Methods of application of breathing gas, i.e., supply to the airways:

1. with an MNJI connected to an endotracheal tube or laryngeal mask, tight or open;
2. with a transtracheal needle or catheter;
3. with a catheter inserted into the trachea (bronchus) to monitor the airway pressure;
4. with a bi-nasal cannula;
5. with a face mask.

**Table 1:** Advantages and disadvantages of HFJV [6].

	Advantages	Disadvantages
**Physiological**	↓ the peak pressure in airways	Risk of volutrauma and barotrauma
	↑ cardiac output	Inspired FiO_2_ inaccurate
	↓ ADH, fluid retention	Gas exchange efficiency is less predictable in obesity or chronic obstructive pulmonary disease (COPD)
**Surgical**	Minimal movement of the vocal cords	Possibility of lower airways contamination during ORL surgery
	Improving access to the surgical field	
	Lowering risk during laser surgery	Contamination of expired air with operational debris
	Minimizing the excursions of the respiratory system	
**Anesthesia**	Advantageous during respiratory tract operations and bronchoscopy	Impossibility of inhalation anesthesia
		Intermittent measurement of ETCO_2_
	Useful in emergencies; transtracheal approach	Necessary humidification
	Minor gas leak at broncho-pleural fistula surgery	High gas flow
		Measured airway pressure inaccurate

### 2.1. Basic Physical Principles of HFJV Function

**Figure 1:**
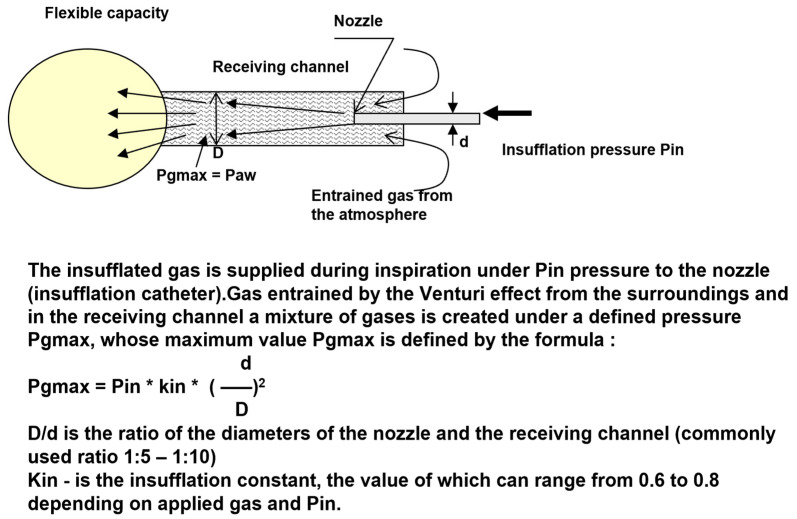
Scheme of the pressure and flow generator with the “nozzle-receiving channel” system [7].

The principle of the HFJV is based on the fact that the catheter introduced into the trachea represents a nozzle located in the receiving channel (trachea), i.e., a generator of gas kinetic power. During the flow of gases through the insufflation catheter, overpressure is created at the distal end of the receiving channel, and the gas flowing through the nozzle creates negative pressure at the proximal end of the generator by the Venturi effect, usually entraining gas from the surrounding atmosphere, which creates a total gas flow through the generator. The pressure (Pg = Paw) and gas flow (Qi) depend on the driving pressure Pin and the ratio of the diameters of the nozzle and the receiving channel (d, D) [6]. The scheme of the pressure and flow generator is presented in Figure 1. For the clinical application, the maximum pressure of the generator PGmax is very important. This value determines the maximum pressure level that can be reached in the lungs at a given insufflation pressure Pin, which can range from 80 to 300 kPa. At the PGmax value, gas does not flow from the lungs or into the lungs, which means that the excess pressure in the lungs, the alveolar pressure (PAI), is equal to PGmax. The value of PGmax is proportional to the value of the insufflation pressure and the square of the ratio of the diameter of the nozzle and the receiving channel (d/D). The constant Kin includes the pressure loss during the flow [3]. 

### 2.2. Catheter form of HFJV with the Original Jet Catheter

In 1967, Sanders developed alternative ventilation to conventional ventilation during micro-laryngoscopy [8]. During passive expiration, the gas was applied above the vocal cords through the application cannula fixed to the laryngoscope. Since that time, several application systems have been developed to provide subglottic, supraglottic, and transtracheal high-frequency ventilation, using different catheters and ventilation frequencies. In 1994, Hunsaker introduced a fluoroplastic, laser-resistant catheter, allowing subglottic ventilation and measurement of airway pressure and end-tidal CO_2_ concentration (ETCO_2_) [9].

**Figure 2:**
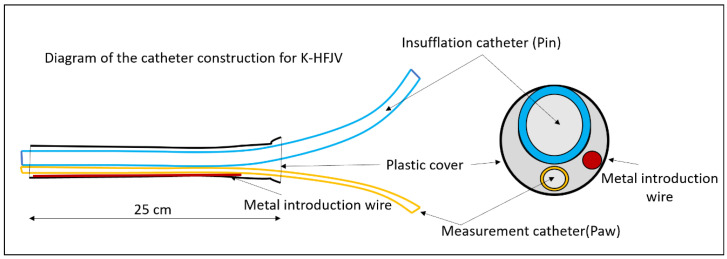
Scheme of the ventilation catheter [7].

**Figure 3:**
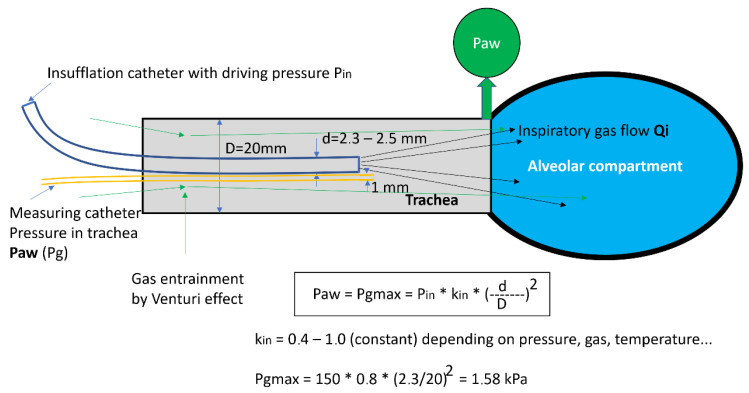
Principles of catheter function in C-HFJV. Paw—pressure in the airways, Pin—insufflation pressure, kin—flow constant, d—diameter of the catheter, D—diameter of the receiving channel (trachea).

The present form of C-HFJV using the original double-lumen ventilation catheter (DVK) is presented in Figure 2. The principles of C-HFJV are shown in Figure 3. In this form of ALV, a ventilation catheter, which consists of an insufflation catheter, a measuring catheter, and a soft metal guidewire used to strengthen the shape of the catheter, covered in the plastic material package, is inserted into the trachea. The catheter, in its original construction, has an outer diameter of approximately 5.5 mm and a length of 27–28 cm.

The catheter is inserted into the trachea, either nasotracheally or orotracheally, usually under direct laryngoscopy. In cases where C-HFJV is applied in thoracic surgery, mainly for procedures on the trachea and bronchi, an insufflation catheter with a diameter of 1.5 mm is usually used for selective ventilation of the lungs. Through insufflation, catheter gas from the ventilator is applied under a pressure of 100–250 kPa. This generates kinetic energy of gases (flow and pressure) during inspiration, as expiration is passive. The measuring catheter serves the purpose of measuring the pressure in the airways. At the same time, it is an active element in the prevention of volutrauma, which in rare cases can put the patient on C-HFJV at risk [6]. Volutrauma and barotrauma are the two potential causes of the same phenomenon–lung injury due to high airway pressure and/or a large distending volume [10]. The ventilator monitors the pressure in the airways, and when the set limit is exceeded, the “total stop” function is activated, and it turns off the ventilator and stops the flow of gases through the Qi insufflation catheter, thus cutting off the flow to the alveolar compartment [11]. The total stop happens if the maximum pressure in the airways, chosen prior by the anesthesiologist, is exceeded to stop possible damage to the lungs. The ventilation frequency is usually 120 c/min, which is a relatively optimal setting in terms of gas exchange.

### 2.3. Advantages of Catheter HFJV

1. Ensures the adequate exchange of blood gases
2. Achieves a free operating field or bronchoscope view
3. Prevents aspiration—the Klein effect. Positive pressure in the space under the tip of the catheter and the flow of gases is continuous during expiration and inspiration from the airways to the atmosphere

### 2.4. Placement of Ventilation Catheter

**Figure 4:**
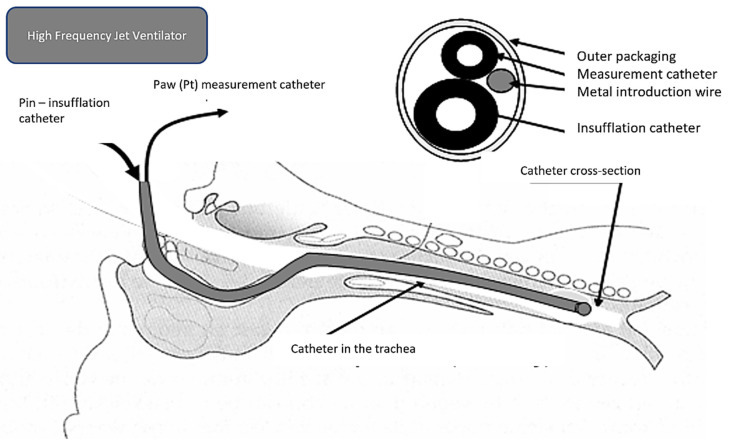
Subglottic placement of the ventilation catheter.

(a) Supraglottic—during surgery in the area above the vocal cords
(b) Transtracheal (very rare, usually as a life-saving procedure
(c) Subglottic (Figure 4.)—introduced to a depth of 6–8 cm below the vocal cords or 3–4 cm above the carina

The advantage of subglottic placement is the provision of ventilation independent of the instrumentation of the physician performing the treatment or diagnostic procedure. Better gas exchange is ensured by the deeper placement of the cannula.

**Figure 5:**
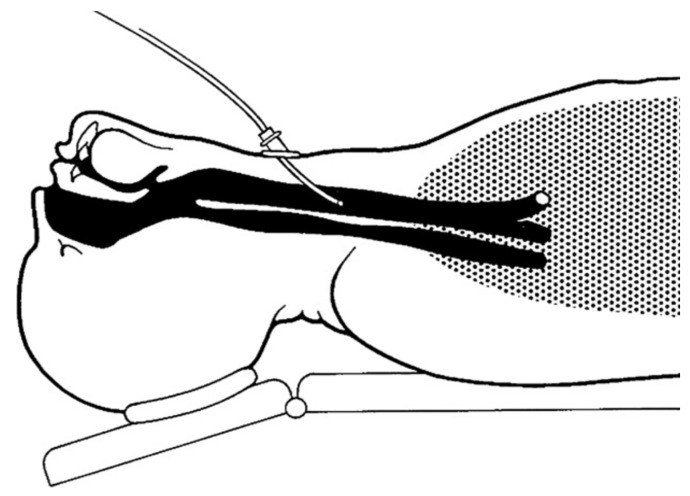
Transtracheal VFDV [10].

However, there is a risk of air trapping in the case of a sudden closure of the vocal cords; therefore, it is not recommended to insert a catheter if the vocal fold is narrowed by more than 50% of the diameter. In this case, it is necessary to place the catheter proximally to the obstruction. In addition to greater safety in cases of airway obstruction, a supraglottically placed catheter ensures ventilation while maintaining a free glottis [12]. The disadvantage is a lower ventilation efficiency, which can be compensated for by increasing the insufflation pressure. In urgent situations, such as the impossibility of intubation or ventilation, it is possible to transtracheally place a thin (1 mm), single-lumen catheter subglottically through the cricothyroid ligament [13]. For HFJV applied in this way, it is essential that the airways are free, without obstruction in the larynx and airways (Figure 5).

### 2.5. Contraindications for the Use of C-HFJV

There are no absolute contraindications for the use of C-HFJV. However, in some cases, it can be a problem to maintain sufficient oxygenation and CO_2_ elimination, especially in patients with morbid obesity or obstructive or restrictive lung diseases [4].

Insufficient oxygenation, manifested by a serious increase in lactate [14], can be corrected by increasing FiO_2_ and subsequently by increasing the insufflation pressure. The next step is to increase the inspiratory pressure, prevent respiratory entrainment of air during inspiration, and possibly insert the catheter more distally into the airway.

Insufficient elimination of CO_2_ occurs mainly in patients with COPD and obese patients. The solution is to increase the inspiratory pressure to the maximum tolerable level (4.0–4.5 kPa).

### 2.6. Ventilator Settings

HFJV is a form of time-cycled, pressure-limited ventilation (similar to pressure-controlled PCV ventilation). If the ventilator settings are constant, in the case of a decrease in compliance of the lungs and chest, minute ventilation is reduced. The elimination of CO_2_ depends more on the setting of the insufflation pressure than on the frequency of the ventilator.

Tidal volume is not adjustable but is a function of insufflation pressure, lung mechanics, inspiratory time, and physical properties of the catheter. The answer to adjusting the settings can be contradictory. Increasing the rate will cause hypercapnia by decreasing tidal volume if insufflation pressure and inspiratory time remain constant.

Insufflation pressure on the ventilator is set in the range of 120–300 kPa. In adults, Pin is initially set to a value of 150 kPa. By increasing the insufflation pressure, the tidal volume increases, the pressure in the airways increases, and CO_2_ decreases. However, a large increase in insufflation pressure at a high frequency will cause CO_2_ retention because of a short expiratory time. Thus, there is no linear relationship between the insufflation pressure and gas exchange.

Ventilation frequency, i.e., the number of ventilator cycles per minute, represents the span of 12–300 c/min. A higher frequency makes it possible to generate smaller volumes, which results in a smaller amplitude of movement of the vocal cords. The disadvantage of a too-high frequency is the insufficient elimination of CO_2_ and the occurrence of auto-PEEP (positive end-expiratory pressure). For C-HFJV, the cycle frequency is usually set to f = 120 c/min.

Auto-PEEP is a significant component of HFJV, depends on the expiratory time, and increases with frequency and a shorter expiratory time. This increases the air volume in areas of the lungs with a long time constant and a shorter expiration time [6].

Spontaneous respiratory activity in HFJV. The patient can breathe spontaneously (if muscle relaxants are not used) because HFJV does not interfere with spontaneous ventilation. This also enables a smooth transition from HFJV to spontaneous ventilation without the need to intubate the patient or apply some form of an assistor.

Inspiratory time is the time between active insufflation and passive expiration. It is defined as a ratio (percentage) of the insufflation time to the total respiratory cycle, usually set to 50%. Changing the inspiratory time will immediately affect the tidal volume. The shortening results in an increase in auto-PEEP and other difficult-to-estimate changes in gas exchange.

Inspiratory oxygen concentration (FiO_2_) is the result of mixing insufflated oxygen with entrained air. It ranges from 0.4 to 1.0. Increased oxygen concentrations can be dangerous for laser surgery applications, so FiO_2_ should not exceed 40%. With C-HFJV, the average FiO_2_ = 0.5–0.6. (60%).

## 3. Recommendations for the Practice

Based on the author’s clinical experience, simulations of the lungs model, and the results of studies, we would like to provide some recommendations for work with C-HFJV for practice:

1. The C-HFJV method is safe and preferred by surgeons, even in the case of airway obstruction of various degrees and locations.
2. The insufflation catheter must be inserted deep enough (6–7 cm below the vocal cords) and well fixed, usually in the corner of the mouth.
3. It is necessary to maintain a sufficient depth of anesthesia and sufficient relaxation to prevent cough and laryngospasm.
4. Catheter insertion must take place under visual control, as blind insertion can result in dislocation into the esophagus.
5. The position of the catheter should be checked regularly, especially when handling surgical instruments.
6. Before the insertion, in the period in which there is no diagnostic or surgical instrument present, it is necessary to ensure the free passage of expired gases by using an airline.
7. The catheter must contain a measurement channel for measuring airway pressure, as well as a “total stop” system.
8. The methodology requires the cooperation of an experienced anesthesiologist and surgeon.

## 4. Conclusions

Procedures that require a different way of securing the airways or the use of another interface between the ventilator and the patient are problematic because they can cause patient–ventilator asynchrony (PVA) [15]. Be it in patients with airway obstruction, disease complications due to an oncologic process, or viral/bacterial pneumonia, the management is always individualized based on current requirements and issues. Countering these phenomena requires a specific approach and is often a challenge for the anesthesiologist. The decision to use C-HFJV presupposes extensive knowledge of the principles of its operation, consistent selection of suitable patients, and the ability to promptly solve the complications that arise. At the same time, in addition to theoretical knowledge, the anesthesiologist is required to have manual skills, the ability to foresee, have a backup plan, deal with critical situations, and have a sense of teamwork.

Several studies have compared different options for the ventilation of patients undergoing surgical procedures in the area of the larynx, bronchoscopy, dilatation tracheostomy, Fantoni tracheostomy, and thoracic surgery to find the optimal technique for maintaining airway patency, ensuring safe and effective ventilation with optimal access to the operative field [1,6]. But these studies were mostly aimed at evaluating safety and effectiveness [8]. During bronchoscopic treatment of airways in hypoxic patients (bronchopneumonia, CARDS, etc.), the risk of further hypoxia during this type of instrumental procedure is reduced.

Among the most common complications in the past was barotrauma; however, currently, volutrauma is an equally considered issue, as high volumes similarly damage the lungs. These issues were especially present in the early days of HFV. Barotrauma occurred as a result of airway obstruction and blockage of free gas expiration [16]. Unlike manual ventilators, the automatic HFJV has a “total stop” automatic shut-down system in case of exceeding the set pressure limit in the airways; therefore, it is necessary to monitor the pressure in the airways with a measuring catheter. The safety of patients with C-HFJV during the procedure was ensured by the use of adequate monitoring of vital functions [6]. However, the possibility of air contamination by virus-infected cells during C-HFJV remains under discussion. 

Thin insufflation catheters enable much better visualization of the space compared to the endotracheal cannula (6.5–7.5 mm diameter internal dimension), and repeated insertions of the Kleinsasser tube can be avoided. This is especially important in the case of oncologic surgery, where good orientation and uninterrupted surgical performance are essential for the excision of the tumor and its edges free of tumorous cells.

The basic requirement for an anesthesiologist is to ensure high-quality hypnosis, analgesia, amnesia, and muscle relaxation (for surgical procedures). They also require control of the airways, safe ventilation of the lungs, and high-quality monitoring of vital functions. The consumption of anesthetics is usually higher in the C-HFJV group compared to the group of intubated patients due to the effort to ensure deep anesthesia and relaxation as a prevention of laryngospasm. As a result, the duration of anesthesia is usually longer in patients ventilated with C-HFJV [16]. 

In conclusion, we can say that catheter methods of HFJV have proven to be very effective ALV methods in technical experimental conditions as well as in clinical applications. C-HFJV can largely eliminate preoperative mild hypoxia and hypercapnia and contributes to the decrease in ventilator-associated pneumonia (VAP) [17], as the placement of the catheter reduces the possibility of bacterial or viral (including SARS-CoV-2) aspirations from the oropharynx. Using C-HFJV with a double-lumen ventilation catheter for diagnostic procedures as well as surgical procedures can be applied with several HGJV ventilators, such as Paravent PATe, Paravent V from Kalas Medical, s.r.o., or Monsoon from Acutronic.

## Data Availability

Not applicable.
